# Radiographic and histomorphometric evaluation of Autogenous Particulated Dentin for Alveolar Ridge Preservation: A Systematic Review and Meta-analyses

**DOI:** 10.4317/jced.63147

**Published:** 2025-10-17

**Authors:** Enrique Castells-Mira, Ferran Sánchez-Benito, Pedro José Almiñana-Pastor, Andrés López-Roldán

**Affiliations:** 1 Department of Stomatology, Faculty of Medicine and Dentistry, University of Valencia, Valencia, Spain

## Abstract

**Background:**

The present systematic review aimed to identify and summarize the radiographic and histological outcomes of alveolar ridge preservation (ARP) using autogenous particulate dentin (APD) compared with spontaneous healing (SH) or other materials in alveolus post-extraction

**Material and Methods:**

The protocol of this PRISMA systematic review was registered in PROSPERO (CRD42021245740). Clinical trials (CT) fulfilling specific eligibility criteria were included. Screening, data extraction and quality assessment were conducted by two reviewers. Study results were summarized using random effect metanalyses to synthesize the findings of the trials.

**Results:**

Fourteen articles concerning ten Randomized CT were included, involving a total of 304 participants, reporting data from 458 sockets. Most of the studies were considered as "unclear" risk of bias. Metanalyses indicated less horizontal at coronal third (difference in weighted means (WMD) = 2.04 mm; p&lt;0.001) and middle third (WMD = 1.52 mm; p=0.002) bone resorption in ARP with APD compared to spontaneous healing assessed radiographically. However, no statistical significance was reached in the apical third (WMD = 0.33 mm; p=0.435) or in the vertical dimensional changes (at buccal aspect, WMD = 0.19 mm; p=0.789; and at lingual aspect, WMD = 0.44 mm; p=0.271). It was not possible to perform a meta-analysis for histomorphometric.

**Conclusions:**

ARP with APD is an effective therapy to attenuate post-extraction bone resorption and ridge changes, especially in the coronal width of the socket.

## Introduction

Tooth extraction triggers a physiological healing process in the residual alveolar bone that causes a significant reduction in the width and height of the alveolar ridge (1) and can hinder optimal three-dimensional placement of implants and compromise aesthetic and functional results (2). These dimensional changes range according to the literature between a 11 and a 22% loss in the vertical aspect and from a 29 to a 63% loss in the horizontal aspect during the first 6 months (3). Alveolar ridge preservation (ARP) is an effective therapeutic approach to reduce dimensional shrinkage of the socket and preserve hard and soft tissue dimensions after a tooth extraction (4). A recent systematic review analyzing different modalities of ARP compared to spontaneous healing (blood clot) suggested that alveolar preservation minimizes alveolar bone reduction after tooth loss, therefore reducing the probability of requiring second bone regeneration surgeries (5). It has been suggested that autogenous particulated dentin (APD) could be a graft material due to its structural similarity to bone. It has a low crystallization hydroxyapatite inorganic composition and an organic matrix rich in type I collagen similar to the composition observed in bone tissue (6). There are also non-collagenous proteins within this inorganic matrix such as osteocalcin, osteonectin, sialoprotein, phosphoprotein and bone morphogenetic proteins (BMP) that could improve osteoregenerative properties (7 , 8). APD can overcome some of the limitations of graft materials such as the risk of cross-infection (7), the cost and complexity of processing and storage associated with allografts and xenografts, and the limited osteoinductive capacity of the xenograft (9). It does not require a second surgical site for graft harvesting either, assuming less morbidity and a lower resorption rate than bone autograft (10). This systematic review aims to identify and summarize the scientific evidence provided by randomized clinical trials (RCT) on the effectiveness of APD in ARP compared to spontaneous healing.

## Material and Methods

1. Protocol and registration The present systematic review was carried out following the guidelines of the Preferred Reporting Items for Systematic Reviews and Meta-Analyses (PRISMA) statement. The protocol design was designed before the beginning of the study and recorded in PROSPERO (CRD42021245740). 2. PICO Question Based on the acronym PICO, the following question was established: "In healthy patients (P), what is the effectiveness of alveolar preservation with autogenous particulated dentin (I) compared to spontaneous healing (C) in maintaining the dimensions of the socket (O)?" 3. Eligibility criteria The eligibility criteria for this systematic review were organized by the acronym PICOS: (P) Participants. Adults (&gt;18 years of age) who required the extraction of at least one tooth, with the exception of third molars. (I) Intervention. ARP using APD as a filling material without simultaneous implant placement. (C) Comparison. Spontaneous healing or other filler material. (O) Outcomes of interest: Dimensional changes of the ridge measured radiographically horizontally (buccolingual) and vertically (vestibular and lingual/palatal) from the baseline (dental extraction) to the final analysis (minimum 3 months). Histomorphometry. Additionally, the rate of postoperative complications, the need for additional regeneration at the time of the implant placement and patient-reported outcome measures (PROMs) were collected. (S) Studies: RCTs. Only those articles reporting RCTs or Non-randomized clinical trials with a proper parallel arms or split-mouth design were included. Studies had to have recruited patients in need of at least one tooth extraction (exception of third molars). Studies had to have compared APD as a filling material with unassisted healing (control) or with other filling graft material (active control) to be eligible. Included studies had to have reported dimensional radiographic changes of the alveolar ridge during a minimum healing period of 3 months. 4. Sources of information and search strategy A search strategy adapted to each of the following databases was developed based on the PICO question: Pubmed MEDLINE, Cochrane Library, Scopus and Web of Science. No time limit or preference regarding any language was established. The search strategy was carried out using a combination of Mesh terms and free text terms. The last search was carried out on May 26, 2024. This search was complemented by a manual search in relevant journals such as 'Journal of Clinical Periodontology', 'Journal of Periodontology', 'Clinical Oral Implants Research', 'Clinical Implant Dentistry and Related Research', 'European Journal of Oral Implantology, 'Implant Dentistry', 'International Journal of Oral and Maxillofacial Surgery', 'International Journal of Oral and Maxillofacial Implants', 'International Journal of Periodontics and Restorative Dentistry', 'Journal of Dental Research', 'Journal of Oral Implantology', 'Journal of Oral &amp; Maxillofacial Research', 'Journal of Oral and Maxillofacial Surgery', 'Oral and Maxillofacial Surgery' and 'Oral Surgery Oral Medicine Oral Pathology Oral Radiology'. The manual search also included all the bibliographic references of each of the selected articles and systematic reviews published until May 1, 2024 (11 - 14). 5. Study selection Two reviewers (E.C.M. and F.S.B.) independently read the results of the database search by title and abstract. Both reviewers independently read the full text of those studies that could potentially be included in this systematic review. In case of disagreement in the final selection, it was resolved through open discussion between both reviewers and, in case of disagreement, a third co-author (P.A.P.) acted as referee. The PRISMA flowchart provides an overview of the selection process. 6. Data extraction The data of the studies included in the final selection were extracted by one of the authors (E.C.M). The accuracy of the data was corroborated by another author (F.S.B). In the absence of relevant information or ambiguity, the corresponding authors were contacted via email. Population: age, gender, smokers/non-smokers, location of the tooth, characteristics of the socket. Intervention/Comparison: type of intervention, biomaterial used, number of participants per group, number of sockets, flap elevation, APD preparation protocol. Outcomes: type of outcome collected, evaluation method, follow-up time points. Risk of bias. 7. Risk of bias in individual studies The quality assessment of the studies was carried out by two reviewers (E.C.M. and F.S.B.) independently using the Cochrane Collaboration tool (RoB 2 and ROBINS-1) for the evaluation of risk of bias in randomized (15) and non-randomized (16) clinical trials. Disagreement between reviewers was resolved by open discussion and consensus. 8. Data synthesis: subgroup analysis and parallel analysis The data were collected and grouped in tables depending on the material used and the result parameters. A descriptive summary was made and the possible variations of the studies in terms of methodology, characteristics and results were verified. To facilitate data pooling and synthesis, data were grouped based on healing time and the type of graft material used. Horizontal radiographic changes of the alveolar crest were grouped into coronal third, middle third and apical third. Vertical radiographic changes were grouped in lingual and buccal bone. Random effects meta-analyses of continuous outcome data were pooled and expressed as weighted mean differences (WMD) with their 95% confidence intervals (CI). Statistical heterogeneity was assessed by calculating the I index2 (percentage of variability of the estimated effect that can be attributed to the heterogeneity of the true effects) and the corresponding statistical test of nullity of Q. The level of significance was 5% (=0.05). The analyses were carried out using the same software used to carry out the meta-analysis: R 4.3.1 (R Core Team (2023). R: A language and environment for statistical computing. R Foundation for Statistical Computing, Vienna, Austria. URL http://www.R-project.org/).

## Results

**Figure 1:**
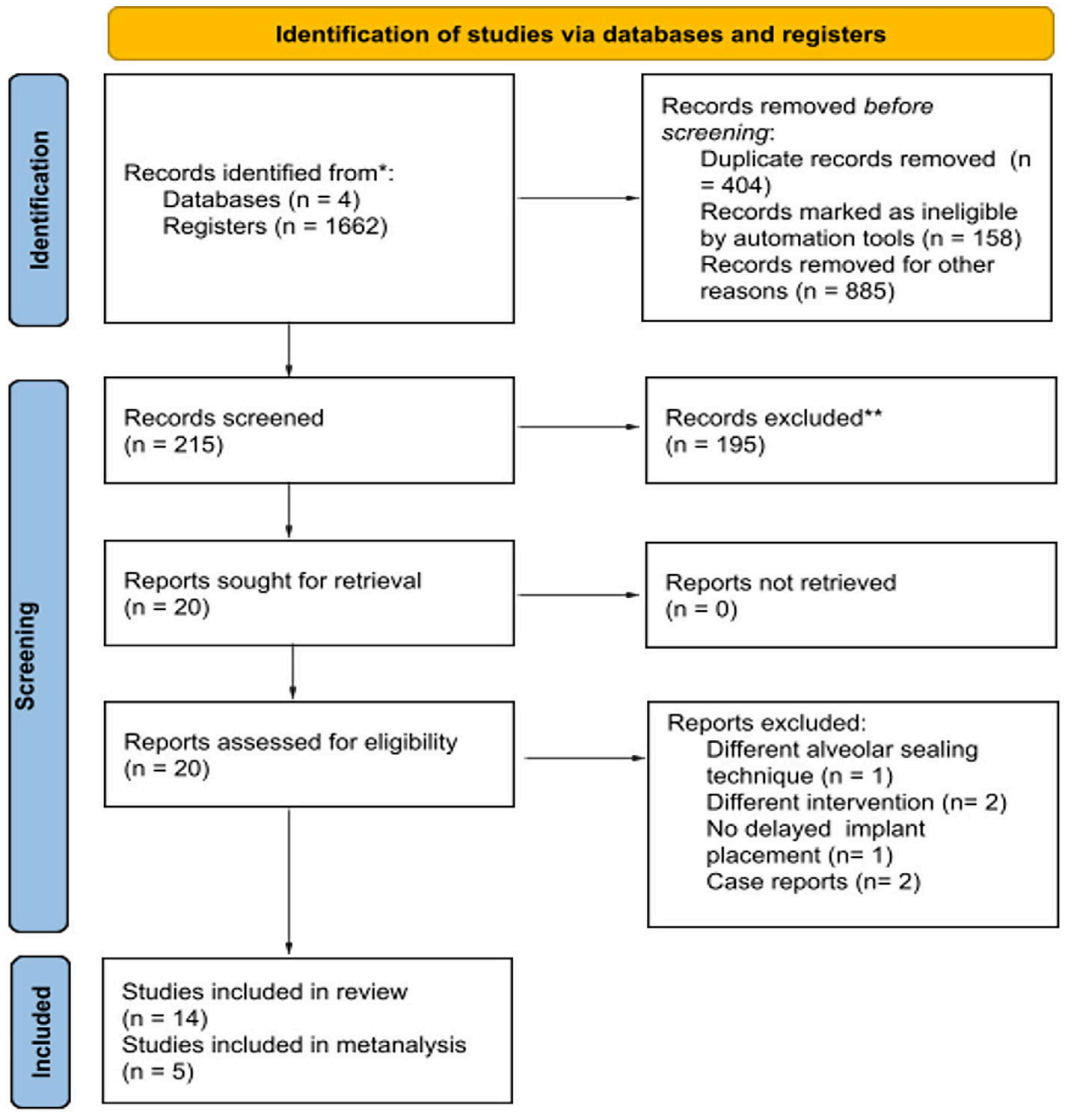
Flow diagram of literature search and selection criteria. Adapted from PRISMA.

1. Study selection The database search identified 1662 references. After excluding duplicates, the total number of entries was 1258. Two additional articles were identified through cross-reference searching. No additional studies were identified in the gray literature search. After phase one, 20 studies were considered potentially eligible. A total of 304 participants from 14 manuscripts were included in phase two, reporting data from 458 sockets (Fig. 1).

1 Screenshot

2. Characteristics of the included studies Table 1 shows the general characteristics of the included articles.

Table 1

2.1 Characteristics of the intervention Five studies did not include smokers (17 - 21), six studies allowed for the inclusion of smokers &lt;10 cigarettes/day (22 - 27) and three studies did not mention whether smokers had been recruited or not (28 - 30). Regarding the anatomy of the sockets, eight studies excluded multirooted teeth (18 - 20 , 23 , 25 , 27 , 28 , 30), three studies included multirooted teeth exclusively(21 , 22 , 26) and both were included in three studies (17 , 24 , 29). Nine studies specified that there was no significant socket damage after the extraction (17 , 19 , 21 - 23 , 25 , 27 , 28 , 30) while two studies specified that the socket showed damage, one of them due to dehiscence in the vestibular table (24) and the other by inclusion of teeth with periodontal involvement with radiographic bone loss &gt;50% of the root length (26). The anatomy of the defect was not specified in three studies (18 , 20 , 29). Whether the flap was elevated at the time of the extraction or not was reported by two studies (19 , 29). Only two studies reported the thickness of the oral tissues in the alveolus pre-extraction, one of them reported the periodontal phenotype (thin/thick) of the different treatment groups (22) and the other reported the thickness of the buccal bone table (26). Regarding flap manipulation, one study reported that it had elevated both flaps to full thickness after the extraction (29) and two clinical trials specified the creation of an envelope flap to anchor the collagen membrane (19 , 25). All the included clinical trials used APD as bone filling material in the experimental group. The origin of the dentin came from the extracted tooth in all the studies, except in one of them, which used the root dentin of third molars (22). The particulate protocol differed depending on the study, using different devices. A Smart Dentin Grinder (SDG) (KometaBio®) was used in seven studies (20 , 21 , 23 25 , 27 , 29), two studies used Tooth Transformer®(18 , 19) and one of them used a BonMaker® device (26). The Korean study (22) used a tooth bank facility (Korean Tooth Bank) that can procure and store teeth, and then process them into bone graft substitutes. The remaining studies used conventional grinders (17 , 28 , 30). The reported particle size ranged between 250-1200µm in all the studies. The disinfection of the APD was carried out using Sodium Hydroxide at a 0.5M concentration, combined or not with ethanol in variable times of 5 to 10 minutes without subsequent demineralization in those articles that followed the SDG protocol. Eight studies used demineralized or partially demineralized APD by lactic acid, hydrochloric acid, tetraacetic acid or nitric acid at different concentrations and times (17 - 19 , 21 , 22 , 26 , 28 , 30) (Table 2).

Table 2

**Figure 2:**
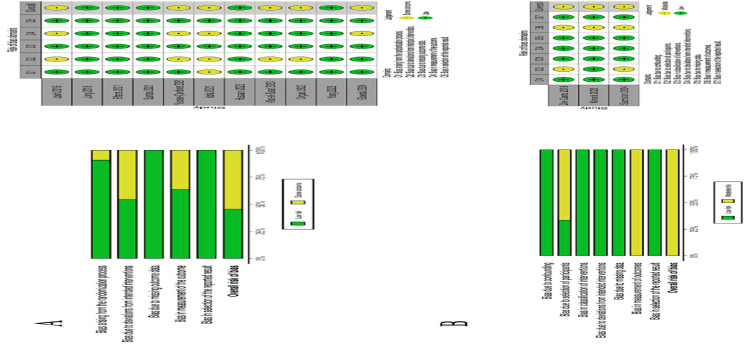
Risk of bias summary of the included studies. (A) Bar graph summarizing the percentage frisk os bias and overall risk of bias per protocol of RCTs; (B) Bar graph summarizing the percentage frisk os bias and overall risk of bias per protocol of non-Randomized.

Intra- and postoperative complications were reported in eight studies. Six studies reported healing without complications (17 , 19 , 21 , 28 - 30) and two studies reported postoperative complications. The first one reported pain in two patients in the APD group and one patient with pain and postoperative bleeding in the xenograft group (22) and the second study reported that five patients in the APD group and three patients in the xenograft+autograft group presented moderate discomfort/pain and postoperative bleeding (25). The remaining six studies did not report data on complications (18 , 20 , 23 , 24 , 26 , 27). Regarding the filler graft material, all the studies used an alveolar sealing method: collagen membrane (17 , 18 , 23 , 27 , 29 , 30), free gingival graft (19) or a combination of both (24 , 25), collagen sponges (22 , 26), platelet-rich fibrin (PRF) membrane (21) or hemostatic sponge (20). One study group combined the socket shield technique with alveolar filling and sealing using a collagen membrane (30). Three intervention groups were established for the descriptive analysis and subsequent data synthesis depending on the filler material used: Test Group: APD. Control Group: Spontaneous healing (SH). Active Control Group: autograft, allograft, xenograft or alloplastics. The healing period prior to the implant placement in the selected studies varied between 3 and 6 months. A healing time of 3 months was reported in two studies (29 , 30), 4 months in ten studies (17 - 23 , 25 - 27) and 6 months in two studies (24 , 28). 3.3 Quality assessment of the included studies According to the Cochrane Collaboration tool for risk of bias assessment (Fig. 2), eleven RCTs were analyzed using the RoB2 tool with a low risk result in five studies (20 , 22 , 24 , 26 , 28) and an unclear risk in six studies (Fig. 2a) (17 , 19 , 21 , 25 , 29 , 30).

2 Screenshot

Three non-randomized clinical trials were analyzed using the ROBINS-I tool, all of them with a result of moderate risk of bias (Fig. 2b) (18 , 23 , 27). In some of the studies it was not clear whether the allocation was concealed or whether participants and staff were blinded where appropriate. 4 Results of individual studies 4.1 Radiographic outcomes All the radiographic outcomes of interest are shown in Table 3.

Table 3

Eleven studies (17 , 20 - 28 , 30) reported the differences between the control and the experimental groups in terms of crestal dimensional changes at horizontal level measured by images obtained by Cone Bean Computed Tomography (CBCT)(17 , 20 - 28 , 30). Vertical linear changes were only reported in seven studies (17 , 20 - 23 , 26 , 27). Three studies did not report radiographic data (18 , 19 , 29). Two studies established the moment after the extraction as the baseline (23 , 27) and the rest of the studies took CBCT prior to the tooth extraction as the baseline. Reference points and lines were used to overlap DICOM (Digital Imaging and Communication in Medicine) files and to calculate crestal dimensional changes at the end of the healing period. For all the radiographic parameters measured linearly in the buccolingual direction, ARP using APD consistently showed more favorable results than the control group. Compared to other graft materials, a comparative study with an alloplastic material (beta-tricalcium phosphate) showed statistically significant better results in the APD group in the buccal-lingual width in the coronal third (p&lt;0.05) (17). One study reported more favorable and significant results in the apical third (p&lt;0.05) in the demineralized APD groups when compared with a xenograft (Bio-Oss Collagen®). Statistical significance was not reached in the rest of the areas (22). One study observed slightly favorable results in the coronal third in favor of the APD group when comparing a xenograft mixture (Cerabone®) and an autograft without observing statistically significant differences (p=0.219) (25). One study compared the mixture of APD with PRF compared to PRF only, observing better levels of buccolingual width in the APD group in the coronal third (p&lt;0.01) (21). For radiographic dimensional changes at height or vertical level, the data obtained in the APD group provided statistically significant and favorable results in three studies when compared with the spontaneous healing control group (17 , 26 , 27), while two studies did not find statistically significant differences (20 , 23). Data reported in the APD groups compared to the xenograft showed no significant differences at a 4-month follow-up (p&gt;0.05) (22). The comparative study of APD with PRF showed favorable and significant results when compared with PRF alone (p&lt;0.01)(21). 4.2 Histomorphometric outcomes Eight studies extracted histomorphometric results (18 , 19 , 22 , 24 - 26 , 28 , 30). A meta-analysis was not possible due to differences in the compared groups, histological methods and time points analyzed. The only study with histomorphometric data from demineralized APD compared with the spontaneous healing showed a percentage of 34.23±13.56% of newly formed bone with dentin compared to 30.22±14.48% obtained from the control group (p&lt;0.05) (19), (Table 4).

Table 4

**Figure 3:**
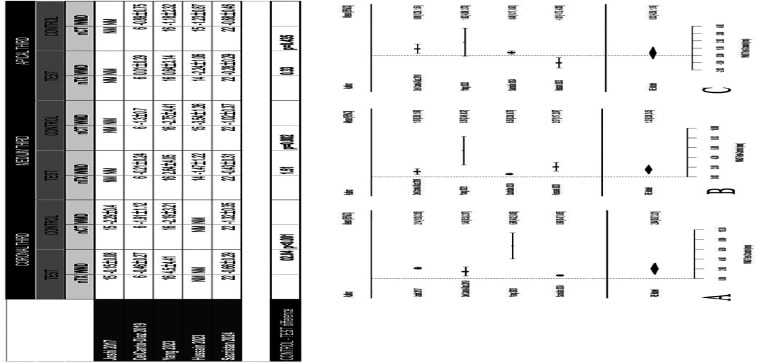
Meta-analyses of horizontal bone changes assessed radiographical by CBCT. (A) Coronal third, (B) medium third and (C) apical third.

**Figure 4:**
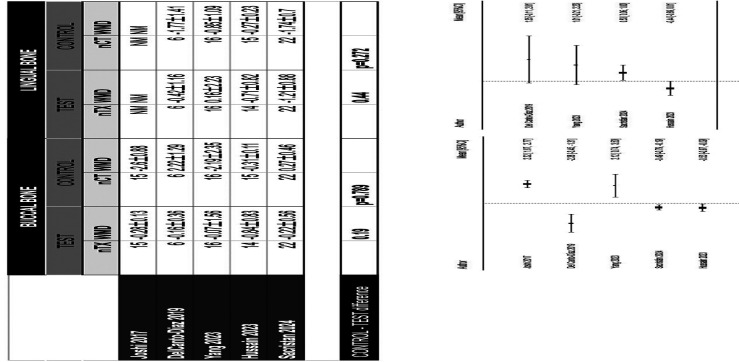
Meta-analyses of vertical bone changes assessed radiographical by CBCT. (A) Buccal bone and, (B) lingual bone.

A comparison between the demineralized APD and a bovine xenograft reported favorable results at 4 months for the group treated with dentin, with values of 32.88±14.48% and 10.72±9.83% for the variables of newly formed bone and residual graft respectively, in comparison with the use of a bovine xenograft, with values of 22±11.01% and 13.20±9.79%, showing differences in favor of the APD group without statistical significance (p&gt;0.05) (22). A similar study at 6 months of follow-up showed more favorable data in the APD group compared to a bovine particulate (Bio-Oss®) in bone neoformation (47.3±14.8% vs. 34.9±13.2%) and in residual particulate (12.2±7.7% vs. 22.1±10.9%) (p&lt;0.001) (24). Two studies compared the histomorphometric results of two different methods of processing autogenous dentin. One study compared the use of dentin from extracted endodontically treated teeth versus non-endodontically treated teeth, without observing significant differences in new bone formation and residual particles (p&gt;0.05) (18). Another histological study compared demineralization or not of the tooth used for ARP, showing more favorable results in the demineralized dentin group at 6 months in terms of resorption of graft particles and newly formed bone (28). 5. Results of quantitative synthesis Five studies (17 , 20 , 23 , 26 , 27) assessed the radiographic dimensional changes of the alveolar ridge comparing spontaneous healing vs. ARP with APD, all with a 4-month healing period (Figs. 3,4).

3 Screenshot 4 Screenshot

It was not possible to establish a statistical synthesis of the comparison with other graft materials. The software used to carry out the meta-analysis was R 4.3.1 (R Core Team (2023). R: A language and environment for statistical computing. R Foundation for Statistical Computing, Vienna, Austria. URL http://www.R-project.org/). 5.1 Change in alveolar ridge width. Coronal Third In the coronal third, a statistically significant reduction of 2.04 mm was observed in favor of the APD-treated group compared to the control group (WMD 2.04; 95% CI 0.87 to 3.21; p&lt; 0.001). Middle Third In the middle third, a statistically significant reduction of 1.52 mm was observed in favor of the test group compared to the control group (WMD 1.52; 95% CI 0.58 to 2.45; p= 0.002). Apical Third In the apical third, a reduction of 0.33 mm was observed in favor of the test group without statistically significant difference with the control group (WMD 0.33; 95% CI -0.50 to 1.16; p= 0.435). A high heterogeneity was detected (p= &lt;0.001; I2 = 89.37%). 5.2 Change in alveolar ridge height. In contrast to the difference observed in width, the effect of the measure in buccal height (WMD=0.19; p=0.789) and lingual height (WMD=0.44; p=0.271) suggested that there is little difference in the vertical reduction of the socket treated with APD without observing statistically significant differences between groups. A high heterogeneity was detected (I2=78,2%).

## Discussion

A statistically significant reduction in the horizontal dimensional changes was observed at 4 months of healing in the coronal and middle portion in favor of the group treated with APD (Coronal Third: 2.04 mm; 95% CI: 0.7 - 3.21 mm; p&lt;0.001; and Middle Third: 1.51 mm 95% CI: 0.58 - 2.45 mm; p=0.002) compared to the spontaneous healing group. This difference is less evident in the Apical Third, in which a reduction of 0.33 mm is observed in favor of the APD-treated group showing no statistical significance (95% CI -0.50 - 1.16; p=0.435). In accordance with these results, ARP with APD could reduce the loss of ridge width in the most critical zone of post-extraction bone resorption. The findings observed in the buccolingual width were consistent with, and even slightly improved, the values reported in previous meta-analyses analyzing other biomaterials used as alveolar filling. In a systematic review and meta-analysis of data from 10 RCTs with a total of 357 participants treated with ARP using a xenograft and resorbable alveolar sealing materials, the most favorable results were observed in the horizontal radiographic changes measured at 1 mm from the crest (MD = 1.84 mm p&lt;0.001) (31). Regarding linear radiographic changes in the height of the alveolar ridge, slightly favorable results were observed in the group treated with APD without statistical significance compared to untreated sockets. The values reported at 4 months of follow-up were a reduction of 0.19 mm (95% CI -1.23 - 1.62 mm; p=0.789) in the buccal area and of 0.44 mm (95% CI -0.34 - 1.22; p=0.271) in the lingual area in favor of the group treated with dentin compared to the control group. These findings were similar to the post-extraction dimensional changes described in the literature after tooth extraction, being less significant than those observed in the buccolingual width of the alveolar ridge (32 , 33). A non-homogeneous distribution of the treated sites was observed in the studies included in the quantitative synthesis. Many studies grouped multi-rooted teeth and single-rooted teeth indistinctly. The post-extraction remodeling process differs in molar and non-molar sites due to the configuration, the size of the socket and the difference in bone phenotype (34). This circumstance may justify the high heterogeneity and dispersion observed in the data analyzed in the statistical synthesis. Histomorphometric data of the included studies revealed that post-extraction sockets filled with APD compared to particulate xenografts of bovine origin showed a greater amount of newly formed bone and a lower percentage of residual graft at 4 months (demineralized APD: 32.88±14.48% and 10.72±9.83% vs xenograft: 22±11.01% and 13.20±9.79%) (22) and at 6 months (APD: 47.3±14.8% and 12.2±7.7% vs. Xenograft: 34.9±13.2% and 22.1±10.9%) (24). These data are similar to the results shown at 6 months of healing in a previous meta-analysis of different graft materials from different sources reporting a percentage of newly formed bone (control 45%; allograft 40%; xenograft 28%; alloplastic 39%) and residual graft material (allograft 20%; xenograft 23%; alloplastic 18%) (35). ARP techniques seemed to be more effective than spontaneous healing in preserving the dimensions and contours of the alveolar ridge, but their benefit was more limited in improving bone formation(36). The observed data seemed to suggest a more rapid bone degradation and neoformation in sites treated with APD than the data reported for sites treated with a xenograft bone particulate and they were comparable to those reported with the use of an allograft. These favorable results in the use of APD could be justified by its physicochemical properties. On the one hand, its high inorganic content (hydroxyapatite) provides a low resorption rate that allows to maintain the volume for bone neoformation (osteoconductive properties) (37 , 38) and on the other hand, during its degradation it can release osteo-regulatory growth factors/proteins (e.g., BMPs) which are integrated/fossilized in the hydroxyapatite crystals that can act by stimulating osteoblastic differentiation(39) and accelerate bone turnover and bone neoformation (osteo-inductive properties) (40). The APD can be prepared using different methods. Three types of APD can be found depending on the protocol and the degree of demineralization: demineralized, partially demineralized or non-demineralized dentin matrix. According to some authors, the degree of demineralization could play an important role in the osteogenic effect. A greater degree of demineralization could increase the surface roughness and exposure of the collagen matrix allowing for a greater cell attachment (41 , 42) and an acceleration in degradation resulting in the early release of fossilized growth factors inside the inorganic structure of dentin which may improve its regenerative capacity (42 - 44).The presence of growth factors such as insulin-like growth factor-II (IGF-II), transforming growth factor beta (TGF-ß) and bone morphogenetic protein (BMP) may be of capital importance during healing(45). Some authors showed the efficacy of a partially demineralized chairside-prepared APD for clinical ARP procedures in humans (46). However, the preparation process of partial demineralization of dentin is lengthy (47); it could reduce the volume of the available graft material (48) and the concentration of growth factors due to critical exposure (49); and it could collapse the three-dimensional structure of the dentin (50). A double-blind parallel-group RCT with a 6-month follow-up comparing demineralized APD vs. mineralized APD reported post-extraction horizontal and vertical radiographic dimensional changes with no statistically significant differences between groups (p&gt;0.05). In the histomorphometric analysis (n=4 per group), greater bone formation and fewer residual particles were observed in the demineralized APD group (48.40±11.56 and 11.45±4.13) than in the mineralized APD group (37.55±8.94 and 17.05±5.58) without statistically significant differences (p&gt;0.05). No postoperative complications were reported in either group (28). This seems to indicate that both APD processes are valid to obtain a favorable result in their use as a graft material. A histological study comparing the use of APD from endodontic teeth with APD from non-endodontic teeth revealed that at 4 months of follow-up there were no detection of significant inflammatory signs, nor presence of endodontic sealing materials and similar percentages of bone neoformation and residual graft particle, with no statistically significant differences between groups (p&gt;0.05) (18). These data seem to indicate that endodontic teeth could also be used as donor teeth for obtaining APD. The use of teeth for APD preparation requires considerable time and effort for cleaning, disinfection and preparation. Its use is conditioned by the presence of a donor tooth with a hopeless prognosis and the required amount to fill the alveolar defect. An in-vitro study revealed that at least 1.0cc of particulate volume can be obtained for every 0.25g of tooth (51). Alternatives to overcome these conditions have been proposed in the literature, such as the use of third molars (22), the use of deciduous teeth 50], preservation of post-extraction teeth for future use (52) or the use of non-autologous dentin as an allograft dentin, a procedure already developed in South Korea (Tooth Bank System) (53 , 54) or xenograft dentin (55). Limitations: This systematic review has certain limitations such as its high heterogeneity and lack of available information. The main limitation is the small number of RCTs with a small sample and with different methods of evaluating results. Despite this, it has been possible to carry out a synthesis of radiographic data that allows us to better understand the dimensional behavior of the APD. Future research on this topic should include properly designed RCTs that follow the most recent version of the CONSORT statement with the objective of evaluating the effect of the use of autologous teeth providing clinical, radiographic, histological evaluations and focused on patient-reported outcomes.

## Conclusions

Within the limitations of this review, ARP with APD following extraction has short-term positive effect on the alveolar ridge. APD could reduce the dimensional alteration of the horizontal ridge by 2.04 mm compared to spontaneous healing at 4 months of healing.

## Figures and Tables

**Table 1:** Characteristics of the included studies.

Author/Year	Study Design	Participants/Sites	Socket anatomy	Smoking	Intervention						Surgical technique	Follow-up	Drop-outs	Method of assessment
					ARP		ARP Comparator		SH					
					Graft material	Sealing material	Graft material	Sealing material	Graft material	Sealing material				
Joshi 2017	RCT Pilot Study	15/45	All sites	No smokers	Autogenous Tooth Graft (ATG)	Allogenic collagen membrane	Beta-tricalcium phosphate (Β-TCP) alloplast	Allogeneic collagen membrane	Blood Clot	Allogenic collagen membrane	Flapless, atraumatic extraction, secondary intention healing	4m	0	CBCT
Jung 2018	RCT	24/24	Molars and premolars	<10 cig/d	Autogenous Demineralized Dentin Matrix (DDM)	Atelocollagen plug	Deproteinized bovine bone with collagen (DBBC)	Atelocollagen plug			Flapless, atraumatic extraction, secondary intention healing	4m	6	CBCT and Histology
Del Canto-Di­az 2019	Clinical Trial Non-Randomized. Pilot Study	6/12	Non molars teeth	<10 cig/d	Autologous Dental Material (ADM)	Collagen membrane 15x20 mm			Blood Clot	Collagen membrane 15x20 mm	Flapless, atraumatic extraction, secondary intention healing	16w	3	CBCT
Minetti 2020	Clinical Trial Non-Randomized. Pilot Study	28/32	Non molars teeth	No smokers	Whole Teeth	Collagen membran	Endodontic treatment whole teeth	Collagen membrane			Flapless, atraumatic extraction, secondary intention healing	4m	0	Histology
Santos 2021	RCT	52/66	All sites	<10 cig/d	Autogenous Mineralized Dentin Matrix (MDM)	Collagen membrane	Xenograft granules	Collagen membrane			Atraumatic extraction, full thickness flap, primary closure	6m	22	CBCT and Histology
Elfana 2021	RCT	20/20	Non molars teeth	NM	Autogenous Whole Tooth (AWTG)	Collagen membrane	Autogenous Demineralized Dentin Graft (ADDG)	Collagen membrane			Flapless, atraumatic extraction, secondary intention healing	6m	0	CBCT and Histology
Yuceer-Çetiner 2021	RCT	9/57	All sites	NM	Undermineralized Autogenous Dentin Graft	Collagen membrane	Undermineralized Autogenous Dentin Graft + PRF	Collagen membrane	Blood Clot	Non Material	Tooth extraction, mucoperiostal flap, primary closure	3m	0	Histology
Isola 2022	RCT	14/28	Non molars teeth	No smokers	Tooth-derived mineralized dentin matrix (DDM)	Free gingival graft			Blood Clot	Free Gingival Graft	Atraumatic extraction, envelope flap, secondary intention healing	16w	0	Histology
Abo-El-Saad 2023	RCT	8/16	Maxillary anterior	NM	Autogenous Dentin Graft + socket shield	Non Material	Beta-tricalcium phosphate (Β-TCP) Alloplast + socket shield	Non Material			NM	3m	0	CBCT and Histology
Hussain 2023	RCT	29/29	Maxillary anterior	No smokers	Autogenous Dentin Biomaterial	Gelatamp			Blood Clot	Non Material	Flapless, atraumatic extraction,	4m	12	CBCT and Histology
Orguić 2023	RCT	37/37	Maxillary anterior	<10 cig/d	Autologous Dentin Graft (ADG)	Collagen membrane + Connective Tissue Graft	Bovine Xenograft + Autologous Bone	Collagen membrane + Connective Tissue Graft			Atraumatic extraction, envelope flap, primary intention healing with conective tissue graft	4m	0	CBCT and Histology
Yang 2023	RCT	32/32	Lower and upper molars	<10 cig/d	Autogenous Partially Demineralized Dentin Matrix (APDDM)	Collagen sponge			Blood clot	Non Material	Flapless, atraumatic extraction, secondary intention healing	4m	15	CBCT and Histology
Sacristan 2024	Clinical Trial Non-Randomized	22/44	Non molars teeth	<10 cig/d	Autologous Dental Material (ADM)	Collagen membrane 15x20 mm			Blood clot	Collagen membrane 15x20 mm	Flapless, atraumatic extraction, secondary intention healing	4m	8	CBCT and Histology
Gowda 2024	RCT	8/16	Lower and upper molars	No smokers	Partially Demineralized Dentin Matrix Block (PDDM block)	Non Material	Partially Demineralized Dentin Matrix Block (PDDM block) + A-PRF	Non Material	A-PRF	Non Material	Flapless, atraumatic extraction, secondary intention healing	4m	0	CBCT

1

**Table 2:** APD treatment protocol. NM = No mentioned.

Author/Year	Donor Tooth	Device	Particle size	Washing Protocol	Desmineralization
Joshi 2017	Extracted Tooth	Grinder convencional	300-500µ	Lactic acid 1N for 15-20 min	Yes (Lactic Acid)
Jung 2018	Third molar roots	AutoBT	500-1000µ	Hidrogen perioxide	Yes
Del Canto-Díaz 2019	Extracted Tooth	SDG	300-1200µ	0.5M NaOH + Etanol 20% for 10 min	No
Minetti 2020	Extracted Tooth	Tooth Transformer	NM	NM	Yes
Santos 2021	Extracted Tooth	SDG	250-1200µ	0.5M NaOH + Etanol 20% for 5 min	No
Elfana 2021	Extracted Tooth	Gold Bone Mill	NM	Basic Ethanol for 10 min /and Hydrochloric acid 0.6N for 30 min	Yes (Hydrochloric acid 0.6N)
Yuceer-Çetiner 2021	Extracted Tooth	SDG	300-1200µ	0.5M NaOH + Etanol 20% for 10 min	No
Isola 2022	Extracted Tooth	Tooth Transformer	1000µ	NM	Yes
Abo-El-Saad 2023	Extracted Tooth	Grinder convencional	300-1200µ	Ethanol 70% and Paracetic Acid 5% for 10 min	SÃ­ (Nitric Acid 2% for 20 min)
Hussain 2023	Extracted Tooth	SDG	300-1200µ	0.5M NaOH + Etanol 20% for 5 min	No
Oguić 2023	Extracted Tooth	SDG	300-1200µ	0.5M NaOH + Etanol 20% for 5 min	No
Yang 2023	Extracted Tooth	Bonmaker	425-1200µ	Hydrochloric acid 3% + Hidrogen Perioxide 10% + Acohol 70% for 30-40 min	Yes
Sacristan 2024	Extracted Tooth	SDG	300-1200µ	0.5M NaOH + Etanol 20% for 10 min	No
Gowda 2024	Extracted Tooth	SDG	300-1200µ	0.5M NaOH + Etanol 20% for 10 min	Yes (EDTA 2 min)

2

**Table 3:** Table Radiographic outcomes. All parameters measured in milimeters. C: Control; AC: Active Control; T: Test; APD: Autogenous Particulated Dentin; SS; Socket Shield; A-PRF: Advanced Platelet-Rich Fibrin, Dash = No data

Author/Year	Groups	CBCT																			
		Change in Horizontal Ridge Width												Change in Vertical Ridge Height							
		Coronal Third				Medium Third				Apical Third				Buccal Bone				Lingual Bone			
		Baseline	4 months	6 months	Difference	Baseline	4 months	6 months	Difference	Baseline	4 months	6 months	Difference	Baseline	4 months	6 months	Difference	Baseline	4 months	6 months	Difference
Joshi 2017	C: SPONTANEOS HEALING	NM	NM		-2,29±0,40									NM	NM		-2,60±0,88	NM	NM		NM
	T: APD	NM	NM		-0,15±0,08									NM	NM		-0,28±0,13	NM	NM		NM
	AC: ALLOPLASTIC (Β-TCPTCP)	NM	NM		-1,45±0,40									NM	NM		-1,72±0,55	NM	NM		NM
Jung 2018	AC: XENOGRAFT (BIO-OSS Collagen)	10,68±2,41	9,00±2,76		-1,68±1,11	11,84±2,28	10,60±2,61		-1,24±0,65	12,71±2,27	12,20±2,24		-0,50±0,19	8,34±2,50	7,21±2,05		-1,14±0,81	8,34±2,54	7,70±2,52		-0,65±0,37
	AC: DEMINERALIZED APD	12,52±1,68	11,74±1,57		-0,78±0,41	14,29±1,84	13,80±1,80		-0,49±0,47	15,94±1,88	15,74±1,89		-0,20±0,15	9,18±2,14	8,21±1,88		-0,97±0,39	9,26±2,09	8,50±2,06		-0,76±0,29
	T: DEMINERALIZED APD +rhBMP2	12,14±1,42	10,60±1,90		-1,54±0,74	12,67±1,99	11,87±2,03		-0,79±0,54	13,38±1,80	13,06±1,73		-0,32±0,21	8,78±1,71	7,95±1,73		-0,82±0,36	8,61±1,65	8,12±1,78		-0,50±0,22
Del Canto-Dí­az 2019	C: SPONTANEOUS HEALING	3,23±0,60	1,31±1,63		-1,91	3,29±0,71	1,99±1,27		-1,3	3,23±0,86	2,34±1,42		-0,89	0,11±1,37	2,33±2,38		NM	10,49±2,98	8,72±2,14		NM
	T: APD	3,14±0,61	2,68±0,48		-0,46	3,15±0,49	2,94±0,54		-0,21	3,22±0,63	3,23±0,65		0,01	0,39±0,83	0,23±20,73		NM	9,50±2,63	9,08±2,16		NM
Minetti 2020	C: APD (ENDODONTIC TEETH)	-	-	-	-	-	-	-	-	-	-	-	-	-	-	-	-	-	-	-	-
	T: APD	-	-	-	-	-	-	-	-	-	-	-	-	-	-	-	-	-	-	-	-
Santos 2021	C: XENOGRAFT (BIO-OSS)	-	-	-	-	-	-	-	-	-	-	-	-	-	-	-	-	-	-	-	-
	T: APD	-	-	-	-	-	-	-	-	-	-	-	-	-	-	-	-	-	-	-	-
Elfana 2021	C: DEMINERALIZED APD					7,86±1,16		NM	-0,85±0,38					9,25±1,9		NM	-0,61±0,20	9,53±1,76		NM	-0,66±0,31
	T: APD					8,11±1,3		NM	-1,02±0,45					8,95±1,6		NM	-0,72±0,27	8,86±1,54		NM	-0,56±0,24
Yuceer-Çetiner 2021	C: SPONTANEOUS HEALING	-	-	-	-	-	-	-	-	-	-	-	-	-	-	-	-	-	-	-	-
	T: APD	-	-	-	-	-	-	-	-	-	-	-	-	-	-	-	-	-	-	-	-
	T2: APD + PRF	-	-	-	-	-	-	-	-	-	-	-	-	-	-	-	-	-	-	-	-
Isola 2022	C: SPONTANEOUS HEALING	-	-	-	-	-	-	-	-	-	-	-	-	-	-	-	-	-	-	-	-
	T: DEMINERALIZED APD	-	-	-	-	-	-	-	-	-	-	-	-	-	-	-	-	-	-	-	-
Abo-El-Saad 2023	AC: ALLOPLASTIC (HA+B-TCP) + SS	-	-	-	-	-	-	-	-	-	-	-	-	-	-	-	-	-	-	-	-
	T: APD + SS	-	-	-	-	-	-	-	-	-	-	-	-	-	-	-	-	-	-	-	-
Hussain 2023	C: SPONTANEOUS HEALING					7,94±1,17	4,39±0,70		-3,54±1,26	8,68±1,10	6,48±0,91		-1,23±0,87	11,32±2,83	10,48±2,50		-0,31±0,11	11,53±2,85	10,82±3,04		-0,27±0,23
	T: APD					7,93±0,99	6,46±1,21		-1,47±1,22	9,06±0,97	7,82±0,87		-2,24±1,06	12,16±1,87	11,88±1,88		-0,84±0,83	12,21±2,32	11,94±2,41		-0,71±0,82
Hussain 2023	CA: XENOGRAFT (BIO-OSS) + AUTOGRAFT	7,88±1,34	6,64±0,85		-1,24±0,99																
	T: APD	8,06±1,34	7,18±1,48		-0,88±0,76																
Yang 2023	C: SPONTANEOUS HEALING	5,98±5,60	NM		-2,19±3,21	12,79±3,72	NM		-2,75±4,41	14,63±3,07	NM		-1,18±2,32	7,19±2,59	NM		-2,19±2,35	6,81±2,68	NM		-0,85±1,09
	T: DEMINERALIZED APD	5,75±5,97	NM		4,50±4,41	9,85±5,94	NM		2,64±4,05	13,52±3,90	NM		0,64±3,14	7,24±2,10	NM		-0,07±1,56	5,29±1,95	NM		0,16±2,23
Sacristan 2024	C: SPONTANEOUS HEALING	4,22±0,74	2,90±0,81		NM	4,00±0,77	2,98±0,85		NM	3,90±0,92	3,22±1,06		NM	1,57±1,04	1,84±1,02		NM	11.90±1,45	10,16±1,61		NM
	T: APD	4,17±0,65	3,51±0,63		NM	3,97±0,71	3,54±0,60		NM	3,72±0,58	3,44±0,67		NM	1,51±1,12	1,29±0,74		NM	11,96±1,97	10,75±1,56		NM
Gowda 2024	AC: A-PRF	10,87±0,5	9,42±0,6		NM	12,98±1,3	11,36±1,0		NM	15,02±1,2	13,04±0,5		NM	10,78±0,9	8,55±0,8		NM	10,70±0,8	8,86±0,8		MM
	T: A-PRF + APD	11,07±0,6	11,36±0,7		NM	13,19±1,0	13,51±1,2		NM	14,96±1,3	15,41±1,6		NM	11,11±1,1	10,87±1,1		NM	11,18±0,9	10,86±1,0		NM

3

**Table 4:** Table Histomorphometric outcomes. All parameters measured in percentages (%). C: Control; AC: Active Control; T: Test; APD: Autogenous Particulated Dentin; SS; Socket Shield; A-PRF: Advanced Platelet-Rich Fibrin, NM: No mention, Dash = No data.

Author/Year	Groups	Histomorfometry		
		VITAL BONE	RESIDUAL GRAFT	CONNECTIVE TISSUE
Joshi 2017	C: SPONTANEOS HEALING	Subjectives Observations		
	T: APD			
	AC: ALLOPLASTIC (Β-TCP)			
Jung 2018	AC: XENOGRAFT (BIO-OSS Collagen)	22±11,01%	13,20±9,79%	64,80±10,11%
	AC: DEMINERALIZED APD	32,88±14,48%	10,72±9,83%	56,40±8,58%
	T: DEMINERALIZED APD +rhBMP2	39,09±15,30%	11,02±12,72%	49,88±11,14%
Del Canto-Díaz 2019	C: SPONTANEOUS HEALING	-	-	-
	T: APD	-	-	-
Minetti 2020	C: APD (ENDODONTIC TEETH)	39,16±11,51%	17,4±7,09%	NM
	T: APD	36,68±8,9%	19,7±13,75%	NM
Santos et al 2021	C: XENOGRAFT (BIO-OSS)	34,9±13,2%	22,1±10,9%	42,9±9,6%
	T: APD	47,3±14,8%	12,2±7,7%	40,5±17,6%
Elfana 2021	C: DEMINERALIZED APD	48,4±11,56%	11,45±4,13%	40,15±7,73%
	T: APD	37,55±8,94%	17,05±5,58%	45,4±4,06%
Yuceer-Çetiner 2021	C: SPONTANEOUS HEALING	-	-	-
	T: APD	-	-	-
	T2: APD + PRF	-	-	-
Isola 2022	C: SPONTANEOUS HEALING	30,22±14,48%	-	29,23±10,16%
	T: DEMINERALIZED APD	34,23±13,56%	19,61±11,49%	27,36±9,65%
Abo-El-Saad 2023	AC: ALLOPLASTIC (HA+B-TCP) + SS	51,4% ± 18,0%	NM	NM
	T: APD + SS	74,9% ± 9,0%	NM	NM
Hussain 2023	C: SPONTANEOUS HEALING	-	-	-
	T: APD	-	-	-
Oguić 2023	CA: XENOGRAFT (BIO-OSS) + AUTOGRAFT	69,61± 13,53%	12,31±7,83%	18,07±6,93%
	T: APD	72,55±12,14%	10,61±5,37%	16,84%±9,18%
Yang 2023	C: SPONTANEOUS HEALING	NM	NM	NM
	T: DEMINERALIZED APD	39,67%	23,66%	36,16%
Sacristan 2024	C: SPONTANEOUS HEALING	-	-	-
	T: APD	-	-	-
Gowda 2024	AC: A-PRF	-	-	-
	T: A-PRF + APD	-	-	-
				

4

## Data Availability

The datasets used and/or analyzed during the current study are available from the corresponding author.
